# Genetic Variants behind Cardiovascular Diseases and Dementia

**DOI:** 10.3390/genes11121514

**Published:** 2020-12-18

**Authors:** Wei-Min Ho, Yah-Yuan Wu, Yi-Chun Chen

**Affiliations:** 1 Department of Neurology, Chang Gung Memorial Hospital, Linkou Medical Center, Taoyuan 33305, Taiwan; drho@cgmh.org.tw (W.-M.H.); lightsky85@hotmail.com (Y.-Y.W.); 2 College of Medicine, Chang Gung University, Taoyuan 33302, Taiwan

**Keywords:** Alzheimer’s disease, cardiovascular disease, cerebral microbleedings, covert infarctions, dementia, single nucleotide polymorphisms, white matter hyperintensities

## Abstract

Cardiovascular diseases (CVDs) and dementia are the leading causes of disability and mortality. Genetic connections between cardiovascular risk factors and dementia have not been elucidated. We conducted a scoping review and pathway analysis to reveal the genetic associations underlying both CVDs and dementia. In the PubMed database, literature was searched using keywords associated with diabetes mellitus, hypertension, dyslipidemia, white matter hyperintensities, cerebral microbleeds, and covert infarctions. Gene lists were extracted from these publications to identify shared genes and pathways for each group. This included high penetrance genes and single nucleotide polymorphisms (SNPs) identified through genome wide association studies. Most risk SNPs to both diabetes and dementia participate in the phospholipase C enzyme system and the downstream nositol 1,4,5-trisphosphate and diacylglycerol activities. Interestingly, AP-2 (TFAP2) transcription factor family and metabolism of vitamins and cofactors were associated with genetic variants that were shared by white matter hyperintensities and dementia, and by microbleeds and dementia. Variants shared by covert infarctions and dementia were related to VEGF ligand–receptor interactions and anti-inflammatory cytokine pathways. Our review sheds light on future investigations into the causative relationships behind CVDs and dementia, and can be a paradigm of the identification of dementia treatments.

## 1. Introduction

With the advance of public health and medicine, the aging population has grown in recent decades; among this population, cardiovascular diseases (CVD) and dementia are the leading causes of disability and mortality [1,2]. The size of the global dementia population was predicted to hit 131 million by 2050. Cognitive and psychobehavioral dysfunctions in people with dementia impose a heavy burden on society [1]. Neuropathology in dementia is commonly heterogeneous, including amyloidopathy, tauopathy, or vasculopathy, and mixed dementia accounts for the major dementia type [3]. Nearly fifty percent of clinically diagnosed Alzheimer’s disease (AD) patients consist of both AD pathology and cerebrovascular disease [4]. Mounting evidence has shown that cardiovascular risk factors (e.g., hypertension, diabetes mellitus, smoking, alcohol, hypercholesterolemia, and hyperhomocysteinemia) and genetics are associated with both CVD and dementia [2,5]. Moreover, cardiac diseases, such as heart failure, atrial fibrillation, or carotid occlusive diseases, may contribute to cognitive impairment through mechanisms associated with reduced cerebral perfusion or cerebrovascular occlusion [6,7]. Studies have found that interactions between the modifiable vascular risk factors and human genome exert extensive effects on the development and deterioration of neurodegenerative diseases [8]. Given that the genetic risk variants are nonmodifiable, prevention and correction of vascular risk factors will help to halt the development of dementia.

There are very few studies that have explicitly explored the genetic relationship between CVD and dementia. Specifically, in the genetic aspect, single nucleotide polymorphisms (SNPs) in different genes have been proven to play causal roles in both CVD and dementia. However, most of the studies recruited participants of the same ethnic origin, thereby rendering the results less prominent [9]. In addition, the interplay between multiple genetic and environmental factors complicates the investigation on the relationship of cardiovascular risk factors and cognitive impairment [10]. Although the evidence levels vary, meta-analyses studies have summarized such findings. A 20-year cohort, which evaluated almost 2 million SNPs in 9317 elderly individuals (>65 years old), revealed that susceptible genetic variants of coronary artery disease, obesity, and type 2 diabetes (T2D) were associated with long-term functional status [11]. Additionally, the Framingham Heart Study reported that participants who had more cardiovascular risk factors or carried at least one Apolipoprotein E (*APOE*) *ɛ4* allele had a greater than two-fold risk of developing dementia [12]. Moreover, aging, *APOE ɛ4* allele, risk alleles in genome-wide association studies (GWAS), and cardiovascular risks increased the 10-year absolute risk of all-cause dementia [8]. These large cohort studies suggested that polygenic variants affecting cardiovascular health status, such as T2D, hypertension, and hyperlipidemia may also influence dementia risks. Thus, we reviewed the literature and summarized a list of genes and SNPs associated with each disease, and undertook further analysis to identify overlapping genes and their associating pathways.

## 2. Materials and Methods

We searched the PubMed database within the time frame from January 1990 to August 2020, and used the following combinations of keywords and Medical Subject Headings (MeSH) terms for a scoping review: “Alzheimer’s disease”, “cerebral autosomal dominant arteriopathy with subcortical infarcts and leukoencephalopathy”, “CADASIL”, “cerebrovascular disease”, “cerebral microbleeds”, “cerebrovascular risk factor”, “cerebral small vessel disease (SVD)”, “cerebral SVD”, “cognition”, “dementia”, “genome-wide association studies”, “GWAS”, “neurodegenerative disorder”, “silent/covert lacunar infarctions”, “single nucleotide polymorphisms”, “SNP”, “vascular dementia”, “white matter hyperintensities”, and “white matter lesions”. Besides the original clinical research studies, we also included randomized controlled trials and meta-analyses. Moreover, important literature cited in the article references were also reviewed if appropriate. No language restrictions were applied.

After extracting the SNPs and associated genes from the selected manuscripts, we categorized and summarized these genetic variants which were shared by each cardiovascular risk factor and dementia, for example, genes that were reported to be influential in causing cognitive decline in diabetic patients. We aimed to explore important genes that are pivotal to disease pathways and thus included high penetrance genes and SNPs from GWASs for pathway analysis by using the Reactome pathway database (https://reactome.org/) [13]. The Reactome Knowledgebase was curated by experts of different domains and was formalized into the database structure. The Reactome entities included nucleic acids, proteins, complexes, vaccines, and small molecules. All the networks were faithful to the primary literature and were further grouped into pathways [13]. We input the gene lists extracted from our review into Reactome and restricted the analysis to human beings. After obtaining the metabolic pathways from Reactome, we inspected the results and summarized the specific pathways according to each cardiovascular risk factor.

## 3. Results

### 3.1. Genetic Variants Connecting Cardiovascular Risk Factors and Dementia

The genetic variants associated with cardiovascular risk factors are heterogeneous, and no single gene or polymorphism can explain the whole picture. Among the variants investigated, the most influential cardiovascular risk to dementia was T2D-related genes.

#### 3.1.1. Diabetes Mellitus

We found 9568 articles discussing T2D and SNPs, among which 53 articles focused on cognitive decline or AD. After reviewing these articles, we retained 10 representative articles and excluded similar ones.

T2D has been recognized as a risk factor of CVD, cerebrovascular disease, and dementia, and the underlying genetic mechanisms remain unknown. However, T2D does not fully explain the development of dementia in that other causal factors, such as amyloid β plaques, tau tangles, cerebral amyloid angiopathy, cerebral SVD, or chronic cerebral perfusion insufficiency also contribute to the pathophysiology of cognitive decline [14]. A GWAS analyzing AD and T2D found that both diseases shared 395 SNPs involved in immunity, cell signaling, cellular processes, and neuronal plasticity (Supplementary Table S1) [15]. Furthermore, a meta-analysis reported that eight SNPs, including *TP53INP1* and *TOMM40*, were associated with both AD and T2D (Supplement Table S2), suggesting that common risk genes were shared by AD and T2D [16].

T2D may aggravate cognitive decline in diabetic people [17,18]. In T2D patients with normal cognition, rs17518584 of the *CADM2* gene was associated with poor processing speed, semantic categorization, and executive functions [17]. In the degenerating population, rs391300 polymorphism of the Serine Racemase (*SRR*) gene was associated with T2D and the progression of mild cognitive impairment (MCI) to AD [18]. In addition, the insulin resistance pathway, such as serine/threonine-protein kinase (AKT) phosphorylation, also plays an important role in dementia risk [19]. *AKT1* rs2498786 CC genotype was a risk variant to insulin resistance and AD [20]. Moreover, patients carrying the TT genotype of Transcription factor 7-like 2 (*TCF7L2*), a diabetes-associated gene, in a cardiovascular cohort had poor executive, attention, and processing speed functions compared to those with CC and CT genotypes [21]. Haptoglobin (Hp) protein is encoded at the chromosome 16q22 locus, and *Hp-1* and *Hp-2* are the two alleles on this locus. Haptoglobin can bind free form hemoglobin and thus prevent tissue from oxidative damage [22]. The elderly diabetic African Americans carrying the *Hp 1-1* genotype had significant cognitive decline compared to those who carried other genotypes, indicating that aging may influence *Hp* expression [23]. Besides aging, risk SNPs may interact with other risk factors to compromise cognition. The synergic effect of serum glycated hemoglobin (HbA1c) level, *APOE* gene, and Val(66)Met in the Brain-derived neurotrophic factor (*BDNF*) gene may accelerate the rates of episodic memory decline [24].

In this study, we performed pathway analysis based on genes that we appraised from reviewed literature (Table 1, Supplementary Tables S1 and S2). We found that most of the above genes participate in the phospholipase C enzyme system and the downstream second messengers, inositol 1,4,5-trisphosphate (IP3) and diacylglycerol (DAG) activities. IP3 mediates intracellular calcium storage while DAG activates protein kinase C isoforms. The calcium concentration also triggers calmodulin–calcium interaction, which modulates synaptic plasticity, learning, and memory (Supplementary Table S3) [25]. In addition, triggering receptor expressed on myeloid cells 2 (*TREM2*) gene has been shown to protect neurons from inflammation and to ameliorate memory impairment in AD mice through interacting with the *AKT* gene in the PI3K/AKT/FoxO3a signaling pathway [26]. Moreover, blockage of tropomyosin-related kinase (Trk) receptors type B of the *BDNF* gene from δ-secretase cleavage may prevent production of *β*-amyloid [27]. The above evidence shows that T2D influences the dementia progress through various metabolic pathways.

#### 3.1.2. Inflammation

We searched the PubMed database for articles that investigated the association between genetics and inflammatory mediators in dementia. Sixty-seven articles were initially identified which, after title, abstract and paper review, resulted in six papers.

The role of inflammatory mediators in dementia and its interaction with amyloid plaques have been discussed extensively [28]. Various expressions of cytokines and chemokines, such as IL-1 and TNF-α, were found in neurodegenerative disorders [29]. A study analyzing elderly participants in the Cardiovascular Health Study showed that rs17042917 and rs4251961 variants of the *IL1RN* gene were associated with the baseline cognitive status, supporting the proinflammatory cytokine IL-1 as a biomarker of dementia status [30]. The Paraoxonase (*PON*) gene cluster of lactonases comprises three genes, *PON1*, *PON2*, and *PON3*, on chromosome 7. PONs have a protective effect on the cardiovascular system and are associated with coronary heart disease, familial hypercholesterolemia, T2D, and atherosclerosis [31]. Vascular dementia (VaD) and AD were associated with different genotypes at codon 192 of the *PON1* gene in that homozygous glutamine-glutamine at codon 192 was associated with VaD, whereas homozygous arginine-arginine was more associated with AD [32]. Moreover, replacement of Cys to Ser at codon 311 of the *PON2* gene occurred more frequently in VaD and in *APOE ɛ4* carriers of AD than in the controls (Table 2) [33]. The interaction between systemic inflammation and the brain was achieved mainly by cytokines and inflammatory mediators crossing the blood-brain barrier. Genetic variants associated with these inflammatory factors render more extensive investigations.

#### 3.1.3. Hypertension and Hyperlipidemia

We searched the PubMed database for articles that investigated the association between genetics and hypertension. Thirty-four articles were initially identified and after abstract and paper review, we found five papers discussing cognitive decline or AD in hypertension patients.

Hypertension is one of the major modifiable risk factors leading to dementia [1]. The interaction between blood pressure and genetic variants on cognition has not been revealed. One study showed that individuals with homozygotic Val allele of the *BDNF* gene had better associative memory and faster processing speed than the Met allele carriers, especially in female or hypertensive participants [39]. Another study reported that the T allele of rs17070145 of WW and C2 domain containing one (*WWC1* or *KIBRA*) gene was associated with lower executive function, especially in women with hypertension [35]. A linkage analysis study also showed that serum A*β*40 levels in nondemented hypertensive patients were associated with rs6703170 of the presenilin 2 (*PSEN2*) gene and rs2514299 on chromosome 11q14-21 area [34]. Common diseases may share the same risk genetic variants, for example, by applying family-based association tests, one study found that rs2033610, rs2596164, and rs2278317 variants of the Ryanodine receptor 3 (*RYR3*) gene were associated with risk of hypertension, T2D, and AD [40]. In addition, C-carriers of Clock Circadian Regulator (*Clock*) *T3111C* polymorphism who had hypertension and *APOE ε4* carriers with dyslipidemia had a higher risk of conversion to AD. These results showed that *Clock T3111C* and *APOE* status may interact with hypertension and dyslipidemia to promote the conversion of MCI to AD (Table 2) [36].

We searched the PubMed database for articles which investigated the association between genetics and hyperlipidemia. Twelve articles were initially identified and after abstract and paper review, we found two papers reporting genetic variants that might lower cognitive decline. A microarray study reported that three SNPs (rs12474609, rs10201482, and rs980286) in the Low-density lipoprotein receptor-related protein 1B (*LRP1B*) gene may have a cognition protective effect against AD [38]. Moreover, variants in the Cholesteryl ester transfer protein (*CETP*) gene were associated with lower risk of cardiovascular events and long life [41]. There is a substitution of isoleucine to valine in SNP rs5882 at codon 405 of the *CETP* gene. A prospective cohort reported that individuals with homozygous variants had slower deterioration of memory and a lower risk of developing dementia (Table 2) [37].

### 3.2. Cerebral Small Vessel Diseases and Dementia

Cerebral SVDs are proposed as a surrogate or biomarker of stroke and dementia. The hallmark of cerebral SVD is mainly endothelial changes in the cerebral arterioles and microvessels that present different features on magnetic resonance imaging (MRI), such as white matter hyperintensities (WMHs), cerebral microbleeds (CMBs), covert infarcts (CIs), and large perivascular space [42,43]. Large perivascular spaces were associated with rapid deterioration of information processing speed and a four-fold risk of developing VaD [44]. Various risk factors for cerebral SVD have been reported including age, atheromatous vesicular change, cerebral amyloid angiopathy, T2D, vasculitis, infections, hypertension, and several genetic diseases such as CADASIL (cerebral autosomal dominant arteriopathy with subcortical infarcts and leukoencephalopathy) [45].

CADASIL is a rare autosomal dominant reversible cerebral angiopathy with the symptoms of ischemic stroke, cognitive decline, migraine, and psychiatric disturbances [46]. In a cohort of 276 CADASIL patients, more than 91% of the lacunes were found at the edge of WMH, rendering the possible association between infarction and WMH lesions [47]. Mutations in the *NOTCH3* gene on chromosome 19 are believed to be the main cause of CADASIL [48]. In addition to the *NOTCH3* gene, some minor variants have been proposed to exert polygenic contributions to WMH burden in CADASIL [49]. In common variants, such as in the *APOE* gene, carriers of the *APOE ε4* allele and of homozygous *APOE ε4* genotype had more WMH volume and CMBs, whereas *APOE ε2* carriers had more WMH burden and increased risk of ischemic infarctions [50].

#### 3.2.1. Genetic Variants Connecting White Matter Hyperintensities and Dementia

We found 217 articles investigating WMHs-related SNPs, of which nine articles discussed cognitive decline or AD.

In a monozygotic twin study and population cohort, larger WMH volume was associated with cardiovascular risk factors and poor cognitive functions [51,52]. In a preclinical AD population, WMH volume and *APOE ε4* variant were more associated with cerebral Aβ deposition [53]. However, WMH may also play a role in non-AD dementia. One study reported that, in MCI patients, those who converted into non-AD dementia may have larger WMH, CMBs, and basal ganglion infarcts than those that remained in MCI. Moreover, mesial temporal lobe atrophy was associated with the progression to AD while WMHs were associated with non-AD dementia [54]. In a study of frontotemporal dementia (FTD), symptomatic FTD patients with *GRN* mutation had more WMH load than patients with microtubule-associated protein tau (*MAPT*) and chromosome 9 open reading frame 72 (*C9orf72*) gene mutations. In addition, more WMHs were found in the frontal and occipital lobes than in other brain areas [55]. In a case report study, a homozygous variant of c.847G>T in the HtrA serine protease 1 (*HTRA1*) gene was found in a patient with symptoms of Cerebral autosomal recessive arteriopathy with subcortical infarcts and leukoencephalopathy (CARASIL) and WMHs in MRI [56]. The polygenic risk effect on cognition can be mediated by WMHs and CMBs, and the risk alleles for AD, regardless of whether they reside within or outside the *APOE*-linkage region (19q13), have been influenced by CMBs, WMH, and coronary artery calcification (Table 3) [57]. Pathway analysis did not show profound downstream metabolic networks, partly due to few genes found to be connected with WMHs and dementia (Supplement Table S4). More specific results were that *NOTCH3* and *APOE* genes both participated in the regulation of AP-2 (TFAP2) transcription factor family, and *MTHFR* and *APOE* genes were involved in metabolism of vitamins and cofactors. The AP-2 protein has been shown to function with Phosphatidylinositol clathrin assembly lymphoid-myeloid leukemia (*PICALM*) as an autophagic cargo receptor in the degradation of amyloid *β* clearance [58].

#### 3.2.2. Genetic Variants Connecting Cerebral Microbleeds and Dementia

Seventeen articles were found with CMBs-associating SNPs, of which nine articles focused on cognitive decline or AD.

CMBs are represented as small vessel wall damage with various degrees of *β*-amyloid accumulation, and different vascular risk factors are believed to impose causative effects on cerebral CMBs [59]. While nonlobar microbleeds were associated with cardiovascular mortality, lobar microbleeds were associated with the risk of mortality by stroke [59]. The Rotterdam Study reported that more than four CMBs per subject was associated with cognitive deterioration. They also found that lobar CMBs were associated with impairments in execution, information processing, and memory, while nonlobar CMBs were associated with decline in information processing and motor speed [60].

A GWAS showed that *APOE ε4* alleles were associated with lobar CMB numbers and the rs769449 variant in *APOE* was significantly associated with CMBs [61]. Stroke patients with *ε2* and *ε4* alleles were reported to have more lobar CMBs in one Korean cohort [62]. Moreover, carriers of the *ε4* allele of *APOE* may have more CMBs than carriers of the *ε3*/ε*3* genotype, and the association was stronger in lobar CMBs [63]. Among hypertensive patients aged between 55 and 75 years old, homozygous carriers of the *APOE ε4* allele had more lacunes than heterozygous *ε2*/*ε4* carriers. In addition, individuals with homozygous variant alleles of four SNPs (rs1699102, rs3824968, rs2282649, and rs1010159) within the 3′-end of the *SORL1* gene had more CMBs than other individuals. This relationship between *APOE*, *SORL1*, and CMBs implies that the amyloid theory may play a role in the mechanism of CMBs, especially in hypertensive patients [64].

Several genetic loci other than in the *APOE* region have been connected to CMBs. A GWAS showed that 19 SNPs were associated with CMBs, whereas the rs55738218 of *SLC12A7* was associated with decreased progression risk of CMBs (Table 4) [65]. The TT genotype of the methylenetetrahydrofolate reductase (*MTHFR*) gene and the presence of CMBs were associated with hyperhomocysteinemia in patients with memory impairment [66]. A study of Taiwanese CADASIL showed that *NOTCH3* mutation carriers had more CMBs in the thalamus and temporal lobe with a wide variation in CMB counts. The CMB counts per CMB volume increased as the disease deteriorated, and the CMB counts increased more rapidly in the thalamus, temporal, and frontal lobes [67]. One study found a frame shift variant (c.236_237delAC) in the *CCM2* gene from three siblings with cognition decline, multiple CMBs, and a family history of early onset AD [68]. Moreover, the homozygous *ε4* genotype of *APOE* was found in two siblings and was heterozygous in a third sibling. This study showed that CMBs and dementia may be attributed to the interaction between the *CCM2* variant and the *APOE* genotype [68]. Polymorphic variants of Aldehyde dehydrogenase 2 (*ALDH2*) were found to be associated with alcoholic cardiomyopathy and stroke [69], and hypertensive male carriers of rs671 in the ALDH2*1/*1 genotype were more likely to have CMBs than *ALDH2* *2 allele carriers (Table 4) [70].

Interestingly, pathway analysis showed that *NOTCH3* and *APOE* genes were both involved in Adaptor protein-2 (AP-2) transcription regulation and vitamins, which were the same metabolic pathways found in WMHs (Supplement Table S5). The analysis suggested that metabolic factors, including vitamins, cofactors, signal transduction by growth factor receptors and vesicle-mediated transport, may contribute to CMBs-associated dementia. 

#### 3.2.3. Genetic Variants Connecting Covert Infarction and Dementia

We searched the PubMed database for articles which investigated the association between genetics and CIs. Thirty-six articles were initially identified and after abstract and paper review we found six papers were associated with cognitive decline or AD.

Cerebral CIs are asymptomatic brain infarcts or the symptoms are too minor to draw the patients’ attention [71]. The definition of CIs has not been reached and the inconsistent inclusion criteria has led to a difficulty in appraisal [71]. For instance, the sizes of atrial fibrillation (AF)-induced silent infarcts may vary from 1.1 to 170 mL [72]. One cohort showed that among AF patients with clinical silent infarctions, 15% had large noncortical or cortical infarcts and 18% had small noncortical infarcts. The AF-related large silent infarctions were associated with poor cognitive performance while the small ones were not [73]. Another difficulty of CI research is that CIs are mostly found incidentally and a portion of subjects with CIs may not be diagnosed throughout their life [74]. Moreover, the causative factors associated with CIs were heterogeneous [75]. Thus, we focused on the genetics in small CIs-related dementia.

In addition to coronary artery disease and myocardial infarction, insulin resistance-related genetic variants were also associated with increased risk of CIs [76]. Specifically, GG genotypes of rs3219175 and rs34861192 of the Resistin (*RETN*) gene, encoding a hormone that links obesity to diabetes mellitus, were associated with increased risk of small artery ischemic stroke [77]. The Apolipoprotein A-I (*APOA1*) gene encodes the main protein component of high-density lipoprotein in plasma, which facilitates the function of cholesterol excretion. One study showed that variants of APOA1 were associated with CIs [78]. The Mono-ADP Ribosylhydrolase 2 (*MACROD2*) gene encodes a deacetylase that mediates removal of ADP-ribose from mono-ADP-ribosylated proteins. In addition, mutations of *MACROD2* may cause hypercholesterolemia. A community based GWAS revealed that T allele carriers of *MACROD2* rs2208454 polymorphism and 22 other SNPs with linkage disequilibrium had a lower risk of CIs (Table 5) [79].

Hyperhomocysteinemia has been found to be associated with CIs [89]. In folate metabolism, the Reduced folate carrier (*SLC19A1*) gene encodes the subunit of replication factor C and facilitates folate delivery to cells. One study showed that a polymorphism of the *RFC-1* gene (80A>G) was associated with arterioles and CIs [87]. The *MTHFR* gene encodes a protein that catalyzes homocysteine remethylation to methionine, and mutation of *MTHFR* is known to induce occlusive vascular disease. A study in a Japanese cohort reported that TT genotype carriers of *MTHFR C677T* polymorphism had higher risks of developing CI and WMH, especially in the elderly (>60 years old) [85].

Atherosclerosis and hypercoagulation status may promote arterial thrombosis. The thromboxane A2 receptor (*TBXA2R*) gene regulated platelet aggregation, and the C allele of rs768963 polymorphism of *TBXA2R* was associated with both large artery atherosclerosis and small artery occlusion [88]. The vascular endothelial growth factor (*VEGF*) gene encodes a heparin-binding protein that induces proliferation and migration of vascular endothelial cells and angiogenesis. The C allele of rs2010963 and T allele of rs3025039 of the *VEGF* gene were associated with small-artery occlusions and CIs [90]. Moreover, the C allele of the -604T>C (rs2071559) polymorphism of Kinase insert domain receptor (*KDR*), which encodes the receptor of VEGF, was associated with increased risk of CI in younger (<65 years old) and male patients [84]. In addition, the endothelial nitric oxide synthase (*eNOS*) gene, which is involved in the *VEGF* pathway, is known to promote angiogenesis in coronary arteries and blood clotting. The haplotypes constructed by the *eNOS* SNPs of -786T-4b-894G, -786T-4b-894T, and -786C-4a-894T were significantly different between patients with CIs and the controls (Table 5) [83].

There are other genes that have been reported as susceptible to cerebral SVD (Table 5). Specifically, the deletion/deletion carriers of rs3840963 of Adrenomedullin 2 (*ADM2*), encoding proteins that regulate cardiovascular homeostasis and prolactin release, suffered from higher risk of chronic kidney disease, CI and WMH [81]. In addition, the c.971_982del of rs61767072 of the adrenoceptor-α2 (*ADRA2*) gene was associated with CIs when patients had hypertension and hyperhomocysteinemia, and the c.145A>G (rs1801252) polymorphism of the *ADRB1* gene was associated with small artery occlusion [82]. The c.1378G>T polymorphism of the *α*-adducin (*ADD1*) gene has also been mentioned as risks of CIs and WMHs [80]. Furthermore, mutations in the *PRKCH* gene were associated with cerebral infarction. Protein Kinase C Eta regulates keratinocyte differentiation by activating the MAP kinase cascade. The AA genotypes of rs3783799 and rs2230500 polymorphisms of *PRKCH* were associated with risks of CI in a Japanese population (Table 5) [86].

Pathway analysis revealed that VEGF ligand–receptor interactions and anti-inflammatory cytokines were associated with CI-related dementia (Supplement Table S6). A previous study showed that VEGF in cerebrospinal fluid may have a protective effect toward patients with early AD biomarkers [91]. In addition, inflammatory factors, such as TNF*α*, IL-6, and IL-10, were associated with both early and late-onset AD [92]. The roles of VEGF and inflammatory factors in the AD cascade warranted further investigation.

## 4. Conclusions

In this review, we searched for and summarized the risk genes or polymorphisms connecting cardiovascular risk factors and dementia. Our results delineated the genetic associations between common diseases, such as T2D and cerebral SVD, and neurodegeneration. We also analyzed the potential metabolic pathways that may provide functional explanations to the risk genetic variants. The most substantial results come from diabetes-related polymorphisms that cover a wide spectrum of metabolic background. Genes such as *GNAI2*, *PRKACG*, or *CALM3* participate in the phospholipase C enzyme system and the downstream IP3 and DAG activities. Among the genes associated with both CMBs and dementia, the *NOTCH3* and *APOE* genes were both found to be involved in AP-2 transcription regulation, suggesting that vitamins, growth factors, and vesicle-mediated transport may contribute to CMBs-associated dementia. The *VEGF* and *KDR* genes were related to VEGF ligand–receptor interactions and anti-inflammatory cytokine pathways, which may contribute to CI-related dementia. For cerebral SVD, although not many risk SNPs have been found to date, more comprehensive investigations are warranted. Our review sheds light on future investigations into the causative relationships behind CVDs and dementia, and can be a paradigm of the identification of dementia treatments.

## Figures and Tables

**Table 1:** Genes Associated with both Diabetes Mellitus and Dementia.

**Gene**	**Location**	**Marker ^1^**	rOR *	**Gene–Phenotype Relationships ^2^**	**Ref**
*AKT1*	14q32.33	rs2498786	0.33 (0.16–0.70)	Breast cancer, colorectal cancer, Cowden syndrome 6, ovarian cancer, proteus syndrome	[20]
**Gene**	**Location**	**Marker ^1^**	r*β* *	**Gene–Phenotype Relationships ^2^**	**Ref**
*CADM2*	3p12.1	rs17518584	0.06		[17]
**Gene**	**Location**	**Marker ^1^**	rP *	**Gene–Phenotype Relationships ^2^**	**Ref**
*HP*	16q22.2	Hp 1-1	<0.01	Anhaptoglobinemia, Hypohaptoglobinemia	[23]
*SRR*	17p13.3	rs391300	<0.01		[18]
*TCF7L2*	10q25.2-q25.3	rs7903146	<0.01	Diabetes mellitus, type 2	[21]
*APOE*	19q13.32	ε4	NA *	Alzheimer disease, macular degeneration	[24]
*BDNF*	11p14.1	Val(66)Met	NA	Signaling by receptor tyrosine kinases	[24]

^1^ Marker means mutation or single nucleotide polymorphisms. ^2^ Gene–phenotype relationships from Online Mendelian Inheritance in Man (OMIM). * rOR, reported odds ratio; r*β*, reported *β* value; rP, reported *p* value; NA, statistics not available.

**Table 2:** Genes Associated with Hypercholesterolism, Hypertension, Inflammation, and Dementia.

**Inflammation**					
Gene	Location	Marker ^1^	rOR *	Gene–Phenotype Relationships ^2^	Ref
*IL1RN*	2q14.1	rs17042917, rs4251961	0.47 (0.09–0.85)		[30]
*PON1*	7q21.3	192QQ/RR	NA *	Coronary artery disease, Coronary artery spasm 2, Microvascular complications of diabetes 5	[32]
*PON2*	7q21.3	311 (Cys → Ser)	NA	Coronary artery disease	[33]
**Hypertension**					
Gene	Location	Marker ^1^	rP *	Gene–Phenotype Relationships ^2^	Ref
*PSEN2*		rs6703170	<0.01		[34]
*WWC1*	5q34	rs17070145	<0.01	Memory	[35]
*Clock*	4q12	T3111C	0.03		[36]
**HypercholesteRolemia**					
Gene	Location	Marker ^1^	rOR *	Gene–Phenotype Relationships ^2^	Ref
*CETP*	16q13	rs5882	0.29 (0.10–0.85)	High density lipoprotein cholesterol, Hyperalphalipoproteinemia	[37]
*LRP1B*	2q22.1-q22.2	rs12474609, rs10201482, rs980286	0.63 (0.38–1.03), 0.71 (0.47–1.09), 0.77(0.50–1.18)		[38]

^1^ Marker means mutation or single nucleotide polymorphisms. ^2^ Gene–phenotype relationships from Online Mendelian Inheritance in Man (OMIM). * rOR, reported odds ratio; rP, reported *p* value; NA, statistics not available.

**Table 3:** Genes Associated with both to White Matter Hyperintensities and Dementia.

**Gene**	**Location**	**Marker ^1^**	rOR *	**Gene–Phenotype Relationships ^2^**	**Ref**
*APOE*	19q13.32	ε4 allele	1.24 (1.07–1.43)	Alzheimer disease, macular degeneration, coronary artery disease, hyperlipoproteinemia, lipoprotein glomerulopathy	[50]
**Gene**	**Location**	**Marker ^1^**	rP *	**Gene–Phenotype Relationships ^2^**	**Ref**
*GRN*	17q21.31	Progranulin	<0.01	Aphasia, primary progressive, ceroid lipofuscinosis, neuronal, frontotemporal lobar degeneration with ubiquitin-positive inclusions	[55]
*HTRA1*	10q26.13	c.847G>T	NA *	Macular degeneration, CARASIL syndrome	[56]
*NOTCH3*	19p13.12		<0.01	Myofibromatosis, infantile 2, CADASIL, lateral meningocele syndrome	[49]

^1^ Marker means mutation or single nucleotide polymorphisms. ^2^ Gene–phenotype relationships from Online Mendelian Inheritance in Man (OMIM). * rOR, reported odds ratio; rP, reported *p* value; NA, statistics not available.

**Table 4:** Genes Associated with both to Cerebral Microbleeds and Dementia.

Gene	Location	Marker ^1^	rOR *	Gene–Phenotype Relationships ^2^	Ref
*ALDH2*	12q24.12	rs671	1.93 (1.21–3.06)	Esophageal cancer, alcohol-related, hangover, sublingual nitroglycerin, alcohol sensitivity	[69]
*AMPH*		rs10263645	0.41		[65]
*APOE*	19q13.32	rs769449	NA *	Alzheimer’s disease, macular degeneration, coronary artery disease, hyperlipoproteinemia, lipoprotein glomerulopathy	[62]
*CCM2*	7p13	Frame shift variant (c.236_237delAC)	NA	Cerebral cavernous malformations-2	[68]
*CTNNA2*	2p12	rs1368908	2.21	Cortical dysplasia, complex	[65]
*LINC01361*	1p31.1	rs10493734, rs10782802	0.46, 0.48		[65]
*LINC01362*	1p31.1	rs1144266, rs1144267, rs7411897, rs11163625, rs1348045, rs11163602	0.45, 0.47, 0.47, 0.47, 0.48, 0.48		[65]
*LOC105374287*	3q29	rs12497385	0.51		[65]
*LOC105374510*	4p15.31	rs1850549, rs66690887, rs67159217, rs10027565	2.56, 2.05, 2.05, 1.97		[65]
*LOC107985396*	1p31.1	rs12132310, rs12140057, rs11163585	0.46, 0.47, 0.47		[65]
*SLC12A7*	5p15.33	rs55738218	0.22		[65]
*SORL1*	11q24.1	rs1699102, rs3824968, rs2282649, rs1010159	6.81 (1.79–25.97), 5.90 (1.54–22.70), 6.87 (1.78–26.44), 4.17 (1.18–14.70)		[64]
*SRGAP1*	12q14.2	rs6581525	2.05		[65]

^1^ Marker means mutation or single nucleotide polymorphisms. ^2^ Gene–phenotype relationships from Online Mendelian Inheritance in Man (OMIM). * rOR, reported odds ratio; NA, statistics not available.

**Table 5:** Genes Associated both to Covert Infarcts and Dementia.

Gene	Location	Marker ^1^	rOR *	Gene–Phenotype Relationships ^2^	Ref
*ADD1*	4p16.3	Gly460Trp	1.36 (0.98–1.88)	Hypertension	[80]
*ADM2*	22q13.33	rs3840963	2.4 (1.2–4.7)		[81]
*ADRA2*	10q25.2	rs61767072	2.03 (1.34–3.10)		[82]
*APOA1*	11q23.3	Alleles I to IV	2.56 (1.19–5.53)	Amyloidosis, ApoA-I and apoC-III deficiency, hypoalphalipoproteinemia	[78]
*eNOS*	7q36.1	894G>T	2.27 (1.34–3.83)	Alzheimer’s disease, late-onset, coronary artery spasm 1, hypertension, ischemic stroke, placental abruption	[83]
*KDR*	4q12	-604T>C	1.596 (1.02–2.50)	Hemangioma, capillary infantile	[84]
*MACROD2*	20p12.1	rs2208454	0.76 (0.68–0.84)		[79]
*MTHFR*	1p36.22	C677T	1.72(1.10–2.68)	Vascular disease, homocystinuria, neural tube defects, schizophrenia, thromboembolism	[85]
*PRKCH*	14q23.1	rs3783799, rs2230500	1.83 (1.00–3.38), 1.62 (1.21–2.17)	Cerebral infarction	[86]
*RETN*	19p13.2	rs3219175, rs34861192	1.49 (1.03–2.17), 1.46 (1.01–2.13)	Diabetes mellitus, Hypertension	[77]
*SLC19A1*	21q22.3	80A>G	1.384 (1.03–1.87)		[87]
*TBXA2R*	19p13.3	rs768963	NA *	Bleeding disorder, platelet-type	[88]

^1^ Marker means mutation or single nucleotide polymorphisms. ^2^ Gene–phenotype relationships from Online Mendelian Inheritance in Man (OMIM). * rOR, reported odds ratio; NA, statistics not available.

## Supplementary Materials

The following are available online at https://www.mdpi.com/2073-4425/11/12/1514/s1, Table S1: Genes associated with both diabetes mellitus and dementia, Table S2: SNPs associated with both diabetes mellitus and dementia, Table S3: Pathway analysis of risk genes for both diabetes mellitus and dementia, Table S4: Pathway analysis of risk genes for both white matter hyperintensities and dementia, Table S5: Pathway analysis of risk genes for both cerebral microbleeds and dementia, Table S6: Pathway analysis of risk genes for both covert infarction and dementia.
